# Potential Use of Avocado Oil on Structured Lipids MLM-Type Production Catalysed by Commercial Immobilised Lipases

**DOI:** 10.1371/journal.pone.0107749

**Published:** 2014-09-23

**Authors:** Eduardo Caballero, Carmen Soto, Araceli Olivares, Claudia Altamirano

**Affiliations:** 1 Centro Regional de Estudios en Alimentos Saludables (CREAS), CONICYT-Regional, GORE Región de Valparaíso, Valparaíso, Chile; 2 Escuela de Ingeniería Bioquímica, Pontificia Universidad Católica de Valparaíso, Valparaíso, Chile; Northeast Ohio Medical University, United States of America

## Abstract

Structured Lipids are generally constituents of functional foods. Growing demands for SL are based on a fuller understanding of nutritional requirements, lipid metabolism, and improved methods to produce them. Specifically, this work was aimed to add value to avocado oil by producing dietary triacylglycerols (TAG) containing medium-chain fatty acids (M) at positions sn-1,3 and long-chain fatty acids (L) at position sn-2. These MLM-type structured lipids (SL) were produced by interesterification of caprylic acid (CA) (C8:0) and avocado oil (content of C18:1). The regiospecific *sn*-1,3 commercial lipases Lipozyme RM IM and TL IM were used as biocatalysts to probe the potential of avocado oil to produce SL. Reactions were performed at 30–50°C for 24 h in solvent-free media with a substrate molar ratio of 1∶2 (TAG:CA) and 4–10% w/w enzyme content. The lowest incorporation of CA (1.1% mol) resulted from Lipozyme RM IM that was incubated at 50°C. The maximum incorporation of CA into *sn*-1,3 positions of TAG was 29.2% mol. This result was obtained at 30°C with 10% w/w Lipozyme TL IM, which is the highest values obtained in solvent-free medium until now for structured lipids of low-calories. This strategy opens a new market to added value products based on avocado oil.

## Introduction

Structured lipids (SL) are defined as triacylglycerols (TAG) which have been chemically or enzymatically modified to change the fatty acids composition and/or positional distribution in the glycerol backbone [1]. They have been developed to meet the demands of health-conscious consumers [2]. These definitions cover any fats produced for special functionality or nutritional use, including cocoa butter equivalents, breast milk fat substitutes, some low-calorie fats, oils enriched in essential fatty acids, and margarines or other plastic fat [3]. The production of SL with medium chain (6–12 carbon atoms) and saturated fatty acids (M) in the *sn-1* and *sn-3* positions and with long-chain (14–24 carbon atoms) saturated or unsaturated fatty acids (L) in the *sn-2* position (“MLM-type” SL) has sharply increased due to their unique nutritional properties [4]. These SL have low caloric value and are thus suitable for controlling obesity, fat malabsorption and/or other metabolic disorders [5]. The average caloric density for this family of fats is 5 kcal/g [6]. A comprehensive review of the literature indicate that there should be no toxicological effects associated with the ingestion of MLM-type SL [7] and the safety of commercial MLM-type SL was validated at both animal and human levels [8].

When SL are consumed, M fatty acids in the *sn-1,3* positions are easily hydrolysed by pancreatic lipase, absorbed into the intestines and carried into the liver, where they are metabolised as a quick energy source; thus, they are unlikely to accumulate as body fat and the remaining 2-monoacylglycerols (MAG) with an L fatty acid in the *sn-2* position are readily absorbed [8], [9]. Hence, the interesterification reactions catalysed by lipases are more efficient than the chemical process because they allow *sn*-1,3 regiospecificity, can be performed in free solvent reactions, and occur at lower temperatures. Active lipases in non-aqueous media catalyse esterification, alcoholysis and transesterification reactions [10]. A major benefit of many lipases is their regiospecificity to the *sn-1,3* positions, where the ester group at *sn*-2 remains unchanged [3]. The *sn-1,3*-specific lipases have been used to incorporate M fatty acids, such as caprylic (C8:0) and capric (C10:0) acids, into the TAG of several vegetable and fish oils, resulting in SL with important clinical and nutritional properties [11], [12], [13], [14], [15]. However, the industrial use of lipases has been limited by their high production costs [16], [17]. Immobilisation in solid carriers is a commonly used strategy to improve the operational stability of commercially available biocatalysts [18]. For example, an immobilised lipase from *Rhizomucor miehei*, Lipozyme RM IM, has been used to produce an MLM-type SL from olive oil enriched with caprylic acid (CA) (43% mol) [19]. Lipozyme TL IM from *Thermomyces lanuginosa* has been used for the acidolysis of corn oil with CA in n-hexane; under optimised conditions, 21.5% mol of CA was incorporated [20].

One of the main parameters in these reactions is the TAG or oil used as the substrate. Thus, we evaluated avocado oil as a new source of oleic acid (L). Cold-pressed avocado oil is relatively new in culinary circles. New Zealand, Mexico, Chile, United States and South Africa are the main avocado oil producers. As its production, commercialisation and marketing are relatively recent; there is limited published information for this product [21]. However, avocado oil is thus a potential feedstock for use in both SL interesterification or acidolysis reactions, because it has 60% of oleic acid [22]. Additionally, avocado and olive oils are advantageous because they can be obtained by a cold extraction method, which is an easy, low-cost technology that retains significant amounts of the fruits' bioactive phytochemicals [23]. Moreover, avocado oil presents higher amounts of campesterol, sitosterol and total phytosterols than olive oil [24], which could remain in the SL produced, generating more benefits for consumers. In the present study, we used Lipozyme RM IM and Lipozyme TL IM to produce an MLM-type SL by interesterification of avocado oil with CA (C8:0) in solvent-free media. We investigated the effects of both temperature and enzyme concentration in the incorporation of CA in the avocado oil structure.

## Materials and Methods

### 1 Materials

Chilean extra virgin avocado oil (brand “Casta”) was used in the reactions. Novozymes, USA kindly provided immobilised *sn*-1,3-regiospecific lipases from *R. miehei* (Lipozyme RM IM) and *T. lanuginosa* (Lipozyme TL IM) used in this study. There are no further patents, products in development or marketed products to declare. This does not alter our adherence to all the PLOS ONE policies on sharing data and materials. CA (>98% purity) and acylglycerol standards were purchased from Sigma-Aldrich. Methyl ester of myristic acid (>99% purity) was purchased from Merck. Pierce BCA Protein Assay Kit was purchased from Thermo Scientific. All solvents and reagents were chromatographic or analytical grade.

### 2 Acidolysis Reaction

The substrate mixture consisted of 2 g of avocado oil and 0.65 g of CA (C8:0), corresponding to a 1∶2 molar ratio of avocado oil:free fatty acid (FFA). This molar ratio corresponds to the stoichiometric value needed for interesterification of free fatty acids at *sn*-1 and *sn*-3 positions, as the lipases used were *sn*-1,3-regioselective. In that case, a theoretical 100% of reaction yield must reach 66.7% mol of CA among the total fatty acids. Different amounts of each immobilised lipase (4–10% w/w of total substrates) were used. Reactions were carried out in solvent-free media at 30–50°C in thermostat-capped cylindrical glass vessels with magnetic stirring at 400 rpm for 24 h. To select the best immobilised lipase and reaction conditions, the average % mol of CA at sn-1,3 positions (% CA) and their standard deviations were calculated. The % CA represents the percentage of CA that is esterified at *sn*-1,3 position into the SL resulting from each reaction condition. It was calculated taking into account both the total fatty acid profile of SL and the fatty acid profile of sn-2 position of the SL. The incorporation of CA in the SL resulting from each reaction mixture and CA at *sn*-2 position, were calculated following methods 3, 4, and 5. The CA at positions *sn*-1,3 were calculated regarding Zou and co-workers [25]. Reactions were performed in triplicate.

The % mol CA at *sn*-1,3 was calculated as follows: 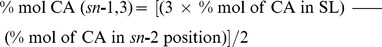



### 3 Analysis of Products by Thin Layer Chromatography (TLC)

The product mixture was separated by thin-layer chromatography (TLC) on silica gel plates and developed with n-hexane/ethyl ether/acetic acid (70∶30∶1.5, v/v/v), as previously described [4]. TLC plates were air-dried and sprayed with a solution of 0.2% (w/v) 2,7-dichlorofluorescein in 95% ethanol and bands were visualised under ultraviolet light at 366 nm. The acylglycerol compounds present in reaction mixtures (triacylglycerols, diacylglycerols and monoacylglycerols) and free fatty acids were identified by comparison with standards. The bands corresponding to TAG or SL were scraped from TLC plates and methylated by the following procedure: Silica gel containing TAG was mixed with 5 mL of methylation reagent (anhydrous methanol/n-hexane/concentrated sulphuric acid; 75/25/1, v/v/v) in a conical flask equipped with a Liebig condenser. This mixture was heated under reflux for 60 min at approximately 80°C. Afterwards, 10 mL of distilled water and 10 mL of petroleum ether were added, and the mixture was transferred to a separating funnel, vigorously agitated and allowed to settle for phase separation. The organic upper layer was recovered, washed twice with distilled water (10 mL) and dried with anhydrous sodium sulphate. Sodium sulphate was removed using a filter paper. The solution was transferred to a flask, and the solvent evaporated by a circulating flux of N_2_. The resulting fatty acid methyl esters (FAMEs) were analysed by gas chromatography (GC) (see point 4 on this section).

### 4 GC Analysis of Fatty Acid Profiles

FAMEs obtained in methodology 3 were dissolved in 100 µL of 0.1% (w/v) methyl ester of myristic acid (C14:0) (internal standard) in n-hexane, and 1 µL of this solution was analysed by GC (Perkin Elmer; USA) equipped with a FID and an Omegawax-320 capillary column (30 m, 0.32 mm i.d., df = 0.50 µm). The injector and detector temperatures were set at 180°C and 250°C, respectively. Helium was used as the carrier gas at a flow rate of 1 mL/min, and the injector was used in split mode at 25 mL/min. The oven temperature program was as follows: 140°C for 2 min, temperature increase to 180°C at 2.5°C/min, a plateau at 180°C for 2 min, temperature increase to 220°C at 3.5°C/min and a final plateau at 220°C for 5 min. The relative FAME content was calculated as a molar percentage on the basis of molecular weight. All analyses were performed in triplicate, and the average values were calculated.

### 5 Pancreatic Lipase-Catalysed sn-2 Positional Analysis

As described by Sahin and co-workers [22], 20 mg of purified pancreatic lipase (porcine pancreatic lipase, crude type II), 1 mL of Tris buffer (pH 8.0), 0.25 mL of bile salts (0.05%), and 0.1 mL of calcium chloride (2.2%) were added to the TAG band previously scraped from the TLC plate. The mixture was incubated in a 40°C water bath for 3 min; 1 mL of 6 M HCl (to stop the reaction) and 1 mL of diethyl ether (to extract the acylclycerides) were added to the reaction, which was then centrifuged at 5000 rpm for 10 minutes. Organic fraction was separated and diethylether was evaporated under nitrogen gas. A 200 µL aliquot was spotted onto a silica gel TLC plate and developed with hexane/diethyl ether/acetic acid (50∶50∶1, v/v/v). The 2-monoacylglycerol (2-MAG) band was visualised under UV light after being sprayed with 0.2% of 2,7-dichlorofluorescein in methanol. The 2-monoolein standard (Sigma) was used for TLC confirmation of 2-MAG reaction products. The 2-MAG band was scraped into a screw-capped test tube, extracted twice with 1 mL of hexane, methylated and analysed by GC as described.

### 6 Transesterification Activity Assay

Taking into account that alcoholysis activity represented on this methodology is not necessary correlated with acidolysis activity (reaction mixture on methodology 2), the alcoholysis reaction was carried out to establish the potential of both commercial lipases tested regarding of their enzymatic activity in organic media. This assay is based on a previously described method [26] that measures the increase in absorbance at 410 nm produced by the *p*-nitrophenol (*p*-NP) released in the transesterification of 30 mM of *p*-nitrophenilpalmitate (*p*-NPP) with ethanol in anhydrous hexane at 40°C. Briefly, 2–4 mg of Lipozyme RM IM or TL IM was added to 1 mL of *p*-NPP solution prepared in anhydrous hexane at 40°C. After 1 minute of stirring at 200 rpm, 60 µL of 96% (v/v) ethanol was added. The release of *p*-NP was monitored every 1.5 minutes for up to 9 minutes. Each sample (20 µL) was transferred into a spectrophotometric cell containing 1 mL of 0.1 M sodium hydroxide and *p*-NP was extracted to the aqueous phase. One unit of activity (U_t_) was defined as the amount of enzyme that catalysed the transesterification of 1 µmol of *p*-NPP per minute under the conditions described above.

### 7 Determination of Protein Content by BCA (bicinchoninic acid)

The Pierce BCA Protein Assay Kit (Thermo Scientific) was used according to the manufacturer's instructions to quantify protein by a colorimetric method. Briefly, 0.1 mL of sample was mixed with 2 mL of work reagent (50 parts of BCA reagent A and 1 part of BCA reagent B from the kit). The tubes were covered and incubated at 37°C for 30 minutes. Samples were cooled at room temperature and the absorbance was measure in spectrophotometer at 562 nm within 10 minutes. The advantage of this method is that it allows measuring protein content in solids, which is relevant given our use of immobilised lipases. For these measurements, suspensions of each immobilised lipase (5 mg/ml) were prepared.

### 8 Statistical Design

To optimise the interesterification reactions, a 3^2^ factorial design was used. The variables were reaction temperature and percentage of enzyme added in the reaction mixture (enzyme concentration). Both variables were selected at three different levels, as indicated in methodology 2. Using this experimental design, 9 interesterification reactions were performed, and the factorial design was evaluated by two-way ANOVA using GraphPad Prism 5.0 software.

## Results and Discussion

### 1 Analysis of Products by TLC

The main compounds were TAG (a mixture of TAG from avocado oil and the product (SL)), DAG, MAG, and FFA.

**Figure 1:**
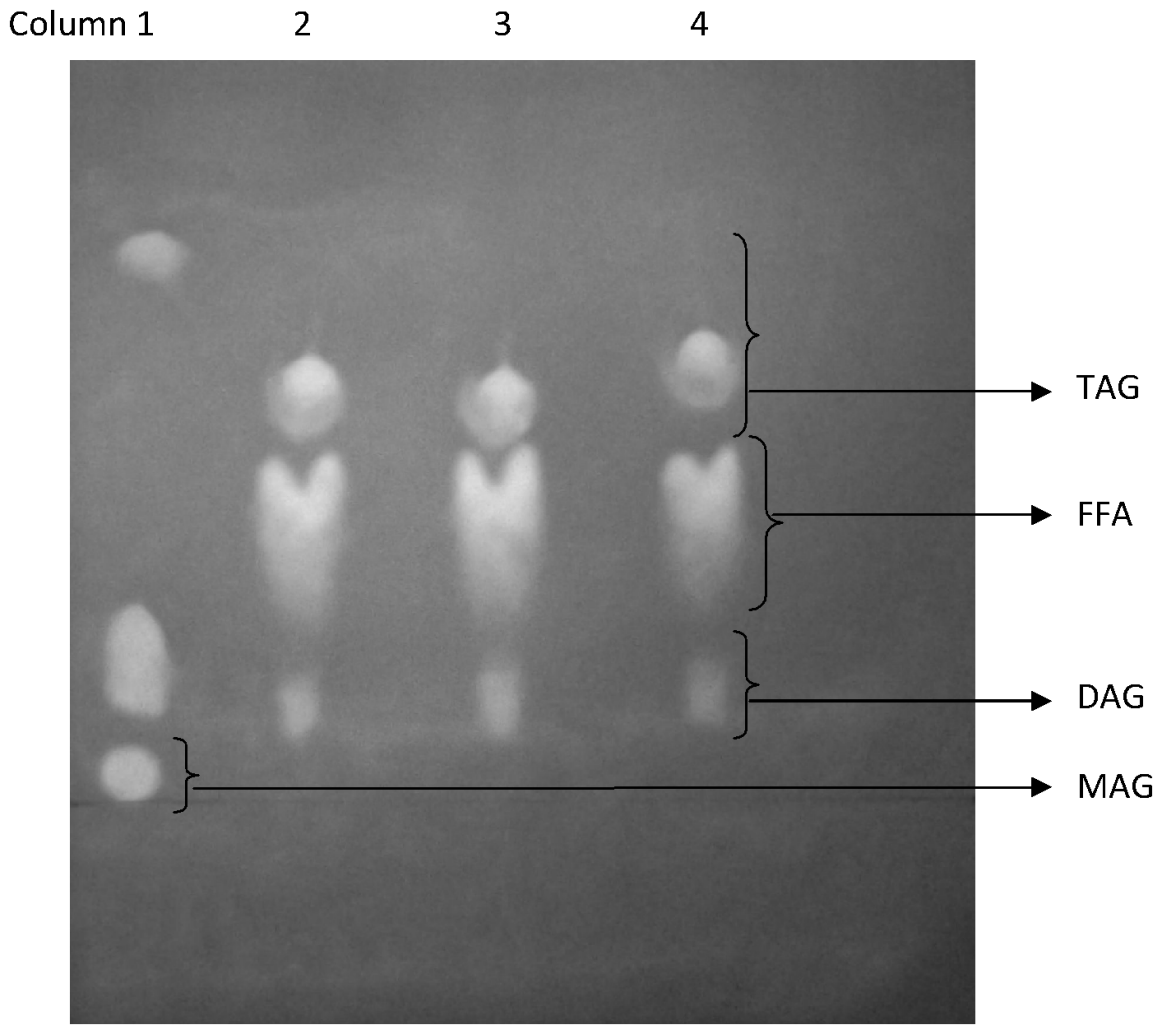
Thin layer chromatography of interesterification reaction samples using avocado oil and caprylic acid as substrates. MAG: monoacylglycerols, DAG: diacylglycerols (mixture of *sn*-1,3 and *sn*-1,2), FFA: mixture of free fatty acids (from avocado oil and caprylic acid), TAG: mixture of triacylglycerols from avocado oil and structured lipids with incorporated caprylic acid. Column 1: monoolein, diolein and triolein standards, Columns 2, 3 and 4: reactions at 30°C with 4%, 7% and 10% of Lipozyme TL IM, respectively.

In Figure 1, columns 2, 3 and 4, representing the enzyme concentration of 4%, 7% and 10% of Lipozyme TL IM, respectively, no MAG were observed. The explanation for this result is related with both the reaction time (24 h), which allowed the conversion of the most of MAG to DAG and TAG, and the lipase mechanism. Thus, if the interesterification mechanism is sequential, the second nucleophilic substitution (*sn*-1or *sn*-3 positions) would occur just after the first one has been substituted with CA, corresponding to an ordered bi-bi mechanism, which is supported by Itabaiana and co-workers [27].

### 2 Incorporation of CA into TAG from Avocado Oil

To quantify the incorporation of CA into TAG from avocado oil, the CA in the original avocado oil was analysed. Table 1 shows the results of GC analysis of the fatty acids profile from avocado oil (column d) in comparison with other researchers (columns a, b, and c). The main results show there is no CA in the fatty acid profile of avocado oil, which allows calculating the incorporation of CA into lipid structure without errors. On the other hand, Table 1 shows that myristic acid (C14:0) can be used as internal standard because it is not present in most of the fatty acid profiles in avocado oil [24], [28]. Other important analysis is the sn-2 fatty acid profile of the avocado oil, which allows determining that oleic and linoleic acids (C18:1 and C18:2, respectively) are the predominant fatty acids at that position (see Table 1). Moreover, when sn-2 of avocado oil and sn-2 of SL are compared, is noted that linoleic acid decreased, indicating that even when the lipase is sn-1,3 regioespecific, can catalysed the acidolysis of some preferential fatty acid at sn-2 position, which is supported by Ihsan and co-workers [29], indicating that inespecific lipases like Lipozyme TL IM, Lipozyme RM IM, and Novozyme 435, showed a selectivity order of C18:3>C18:2>C18:1.

**Table 1:** Fatty Acid Profile from Different Source of Avocado Oil, SL Resulting, sn-2, and sn-1,3 Position of the SL.

Fatty Acids	Avocado oil				d sn-2 avocado oil * (mol%)	SL* (mol %)	sn-2 of SL* (mol %)	sn-1,3 of SL** (%mol)
	a (w/w %)	b (w/w %)	c (w/w %)	d * (mol %)				
C8:0	0	0	0	0	0	19.53±1.2	0.11±0.00	29.24
C14:0	0.06±0.00	0	0	0	0	0	0	0
C16:0	18.74±0.06	16.79	13.47	15.72±0.28	15.33±0.08	12.89±0.75	20.19±0.01	9.24
C16:1	7.98±0.01	10.65	4.53	4.48±0.17	6.85±0.03	2.89±0.10	6.73±0.01	0.97
C18:0	0.51±0.00	0.63	0.32	1.41±0.05	0.96±0.01	0.72±0.02	0.94±0.00	0.61
C18:1	60.56±0.1	47.48	58.83	65.42±0.26	48.41±0.03	45.65±0.89	67.31±0.02	34.82
C18:2	10.87±0.01	14.64	10.86	12.52±0.18	28.38±0.03	8.59±0.66	4.71±0.01	10.53
C18:3	0.61±0.00	1.29	0.81	0	0	0	0	0
C20:1	0.12±0.00	0	0	0	0	0	0	0
C20:3	0.01±0.00	0	0	0	0	0	0	
C20:4	0.01±0.00	0	0	0.45±0.02	0	0	0	

**a**: [24].

**b**: [25]. Standard deviation not reported.

**c**: United States Department of Agriculture, Agricultural Research Service. Available at: http://www.nal.usda.gov/fnic/foodcomp. Standard Deviation not reported.

**d**: Results from Chilean avocado oil, Brand “Casta”.

*: GC analysis of fatty acid profile obtained in the laboratory from avocado oil and SL obtained from the reaction with 10% w/w of TL IM at 30°C.

**: calculated by equation mentioned in methodology 2.2.

### 3 The Effect of Reaction Temperature and Enzyme Concentration on the Incorporation of CA into Avocado Oil

To determine the best temperature and enzyme concentration for interesterification of avocado oil and CA using both lipases studied, the statistical design mentioned in 2.8 was carried out.

**Figure 2:**
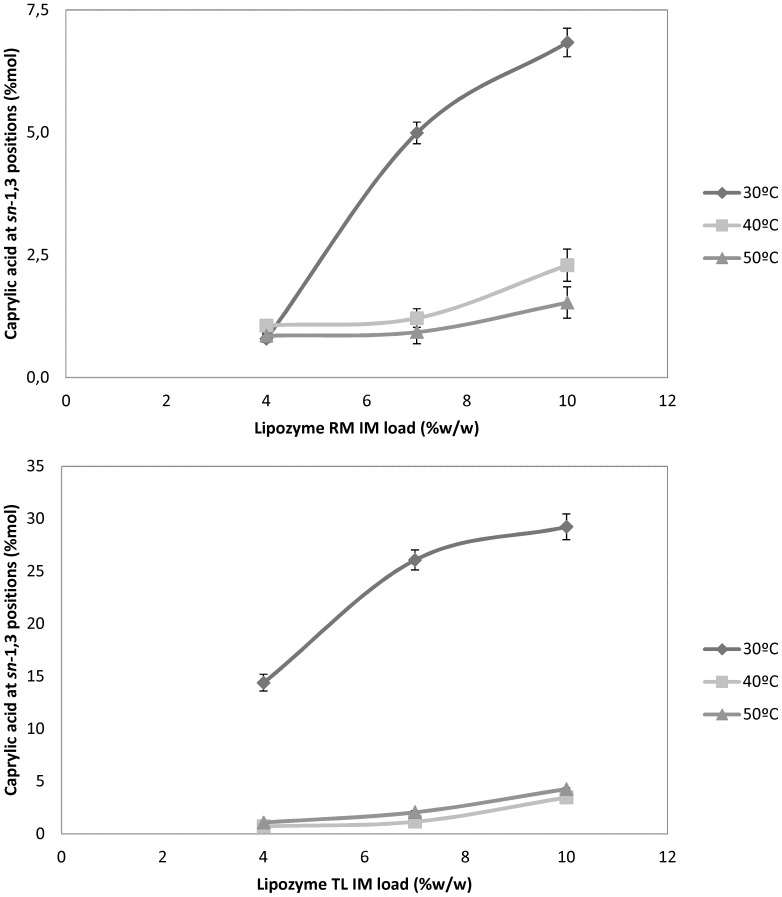
Effect of lipase content and temperature on incorporation of caprylic acid into avocado oil. A) Reactions with Lipozyme RM IM. B) Reactions with Lipozyme TL IM. Each reaction was performed for 24 h at a molar ratio of 1∶2 (TAG from avocado oil: caprylic acid).

The effect of both enzyme concentration and reaction temperature on the incorporation of CA into avocado oil structure to produce MLM-type SL is shown in Figure 2, which displays that CA incorporation was significantly increased (P<0.05) by adding enzyme to the reaction mixture at 30°C. However, the incorporation of CA decreased dramatically from 30°C to 40°C and 50°C with either Lipozyme TL IM or RM IM. Regarding Zhang and co-workers [30] the optimal temperature for Lipozyme TL IM in a solvent system is 35–40°C. Consequently, our results established 30°C as optimal temperature in solvent-free medium, which represents both environmental and energetic advantages. Hence, the main achievement of this investigation is to reach an incorporation value of CA (29.24%) exceeding that the highest reported incorporation (26.9%) [30], which is statistically significant (P<0.05), indicating that avocado oil shows a good potential as feedstock to optimise CA incorporation in the MLM-type SL production as a low caloric fat.

From another point of view, the optimal reaction temperature depends, among others, on the type of immobilisation, chemical modification of enzymes and pH of the reaction mixture [31]. Regarding that, the supports used to immobilise both commercial lipases are different in surface, nature (Lipozyme TL IM and RM IM were immobilised in acrylic resin and anion-exchange resin, respectively) [32], and are based on different types of enzyme-support interactions, providing different characteristics in terms of enzyme thermal stability [33], which is considered an important parameter to scale up the process. Sim and co-workers [34] established that Lipozyme TL IM increases its catalytic activity until 40°C and successive fell beyond this value, presenting thermal inactivation. This result would support both to scale up the process at the optimal temperature of the present study (30°C) without thermal inactivation and to reuse Lipozyme TL IM, increasing the viability of an industrial process.

Table 2 and 3 gives another explanation of the results shown in Figure 2. Table 2 shows the characterisation of each commercial immobilised lipase. The protein content of Lipozyme TL IM is almost 3 times greater than that of Lipozyme RM IM, resulting in more than 3-fold greater enzymatic activity. Taking into account that transesterification activity gives a measurement of the potential synthetic activity in organic media [35], [36], these results could explain the difference observed between both immobilised lipases in Figures 2. Nonetheless, the specific activity (U_t_/mg protein) was similar in both cases.

**Table 2:** Characterisation of Lipozyme TL IM and RM IM.

Biocatalyst	Biocatalyst suspension (mg/ml)	Protein Concentration (µg/ml)	Protein/Biocatalyst ratio (mg/mg)	Enzyme Activity (U_t_/g biocatalysts)	Specific Activity (U_t_/mg protein)
Lipozyme TL IM	5.05	614.5±11.3	121.7	73.5±4.1	0.60
Lipozyme RM IM	5.08	212.2±19.8	41.8	22.5±1.4	0.54

**Table 3:** Comparison of Enzyme Concentration and Total Transesterification Activity.

Biocatalysts	Total transesterification activity (U_t_)		
	4% (1.1 g)	7% (1.9 g)	10% (2.7 g)
Lipozyme TL IM	8.1±0.22	13.9±0.32	19.8±0.35
Lipozyme RM IM	2.5±0.08	4.3±0.10	6.1±0.09

As shown in Table 3, when Lipozyme TL IM and RM IM were compared at any concentration, the values of total activity are 3.24 times greater in Lypozyme TL IM. Comparing the maximum CA incorporation values in Figure 2, Lipozyme TL IM is four times that of Lipozyme RM IM, which indicates a direct correlation between CA incorporation into avocado oil structure at optimal conditions and transesterification activity as a measurement of potential activity of each biocatalysts in organic media.

Although maximal CA incorporation was obtained with 10% w/w, 7% w/w of Lipozyme TL IM was a very plausible alternative to carry out an industrial process. However, an economic analysis is needed to determine if a 3%w/w reduction in enzyme justifies decreasing the incorporation of CA by 2% mol.

These results are supported by Hamam and Shahidi [37], who observed that as the enzyme concentration increased from 2 to 10%, the incorporation of capric acid into single cell oil increased gradually and reached a maximum value (22.4%) at 10% w/w.

On the other hand, most studies that compare immobilised lipases for these types of reactions express enzyme concentration in the reaction mixture as a w/w percentage with respect to the substrate [1], [38] even if is not comparable in terms of total activity. The comparison in mass or % w/w is justified from an operational point of view. For example, Table 3 compares enzyme concentration and total enzymatic activity in the reactions. From these results, we calculated that the maximum value of CA incorporation obtained by Lipozyme TL IM (29.24% mol) could be achieved theoretically with 32.5% w/w of Lipozyme RM IM, but this amount is not convenient for operational and economic reasons.

To support all the above mentioned results of CA incorporation into avocado oil structure under the optimised reaction conditions (30°C and 10% w/w of TL IM), Table 1 shows the results of the fatty acid profile of avocado oil, MLM-type SL produced and its sn-2 position. Regarding the sn-2 position of SL, the most of free fatty acids on this position are long-chain (L) (14–24 carbon atoms) and the acyl migration of CA at position sn-2 was not significant (0.11% mol); moreover, it was lower than Du and co-workers [39], who worked with lipase TL. On the other hand, other researchers investigated the suppression of acyl migration in enzymatic production of SL, concluding that temperature programming is also an strategy to reach this goal [40], which could be consider as a strategy in future proposals to decrease even more the acyl migration. When columns “d” and “SL” on were compared, is noted that CA replaced 19.77% mol of oleic acid (C18:1), which is the more significant fatty acid into avocado oil.

## Conclusions

As a substrate for interesterification reactions with CA, avocado oil showed to be the best alternative respect to other oils to develop a process to produce SL type MLM as a dietary fat. This result highlights an opportunity to optimise this method with currently available reaction media. However, temperature is an important parameter, and its optimum value must be check under 30°C.

The maximum % CA in the SL (29.2%) occurred at 10% w/w of enzyme concentration. However, future studies should integrate economic considerations to determine the optimal parameters for this process.
